# Improving Adherence to Safe Sleep Guidelines for Hospitalized Infants at a Children’s Hospital

**DOI:** 10.1097/pq9.0000000000000508

**Published:** 2022-01-21

**Authors:** Adolfo L. Molina, Meghan Harrison, Candice Dye, Christine Stoops, Erinn O. Schmit

**Affiliations:** From the * Department of Pediatrics, Division of Pediatric Hospital Medicine, University of Alabama at Birmingham, Birmingham, Ala.; † Department of Pediatrics, General Pediatric Residency, University of Alabama at Birmingham, Birmingham, Ala.; ‡ Department of Pediatrics, Division of Academic General Pediatric Medicine, University of Alabama at Birmingham, Birmingham, Ala.; § Department of Pediatrics, Division of Neonatology, University of Alabama at Birmingham, Birmingham, Ala.

## Abstract

**Introduction:**

Sudden unexpected infant deaths are a major problem nationally. We had poor adherence to safe sleep recommendations locally at our institution. Given the significance of this problem, hospital administration at a tertiary children’s hospital tasked a multidisciplinary group of faculty and staff with improving sleep environments for hospitalized infants.

**Methods:**

Our safe sleep task force implemented targeted interventions using the American Academy of Pediatrics policy statement as the gold standard and based on hospital data to address areas of greatest nonadherence to recommendations. We aimed to improve weekly average adherence to 95% over 12 months. A proportions process control chart (p-chart) tracked average weekly adherence over a 52-week time frame. In addition, we performed Student’s t-testing to evaluate differences in adherence over time.

**Results:**

There was a significant improvement in overall adherence to safe sleep recommendations from baseline (M = 70.8%, SD 21.6) to end of study period (M = 94.7%, SD 10.0) [t(427) = −15.1, *P* ≤ 0.001]. Crib audits with 100% adherence increased from a baseline (M = 0%, SD 0) to the end of the study period M = 70.4%, SD = 46) [t(381)= −21.4, *P* ≤ 0.001]. This resulted in two trend shifts on the p-chart using Institute for Healthcare Improvement control chart rules.

**Conclusions:**

Targeted interventions using QI methodology led to significant increases in adherence to safe sleep guidelines. Notable improvements in behavior indicated significant changes in safe sleep culture. We also noted continued adherence in follow-up audits reflecting sustainability.

## Abstract 2

Supplemental Digital Content is available in the text.

## INTRODUCTION

### PROBLEM DESCRIPTION

The United States has an infant (<12 months) mortality rate that is 50% greater than other developed countries (~6 per 1000 births), with approximately 3500 infants dying from sudden unexpected infant death (SUID) annually.^1,2^ SUID is defined as any sudden and unexpected death without an immediate explanation occurring during infancy.^3,4^ It is the leading cause of infant death beyond the neonatal period, with the highest risk in the first 6 months of life.^5,6^ Our state has the highest infant mortality rate in the country (7.89/1000 births), with 449 infant deaths in 2019.^2^ In our county specifically, 10 of 19 infant deaths in 2019 were sleep-related.^7^ The majority of SUID is composed of Sudden Infant Death Syndrome (SIDS) or accidental strangulation and suffocation in bed (ASSB); risks for these are associated with sleep environment and positioning.^8^ Importantly, many of these deaths, particularly accidental strangulation and suffocation in bed, could be preventable with adherence to the evidence-based recommendations of the American Academy of Pediatrics (AAP) Policy Statement.^4^

### AVAILABLE KNOWLEDGE

Multiple institutions have addressed the problem of unsafe inpatient sleep environments through quality improvement (QI). These QI projects used several interventions to improve outcomes, including education of staff^9–14^ and families,^10–12,14–18^ policy changes,^10–13,16,19^ infant specific sleep garments (wearable blankets),^10,13,19,20^ diaper caddies,^20^ and crib cards.^12,21^ However, there is a lack of data regarding interventions in tertiary inpatient pediatric centers outside mother-baby units. Additionally, few of the studies cited above demonstrate the strength of specific interventions or, in tandem, as most were limited to less than two interventions or implemented variably across multiple institutions. We also add to the literature regarding electronic health record utilization to promote safe sleep guidelines in the inpatient setting.

### RATIONALE

**Fig. 1.**
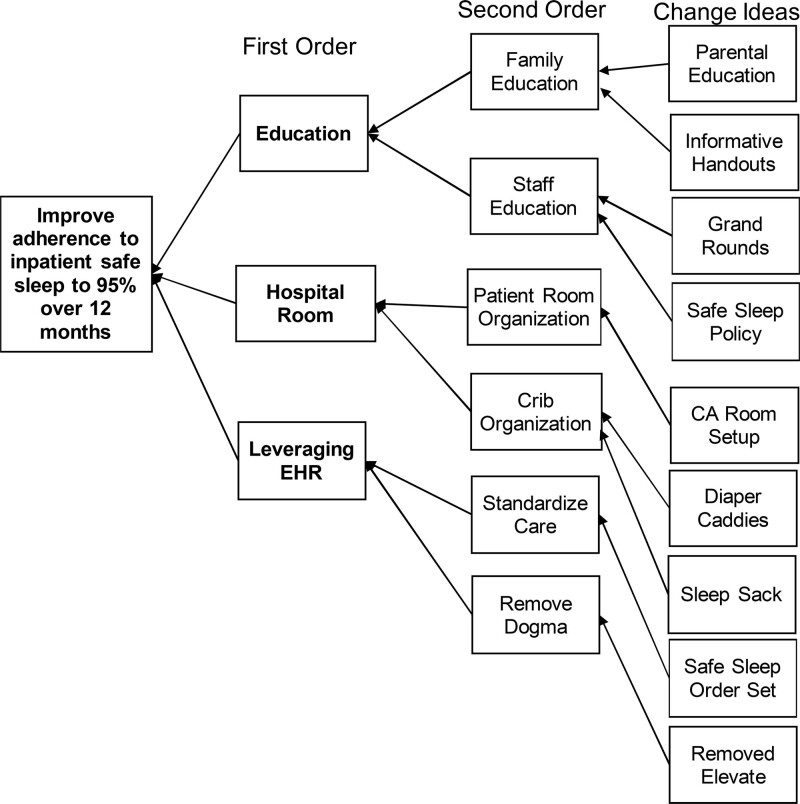
Key Driver Diagram reviewing the three main drivers to our goal to improve adherence to safe sleep. We specifically highlight the importance of education, changing the hospital room environment, and leveraging the EHR through multiple interventions to achieve our goals.

Our institution is an academic, tertiary, non-birthing hospital that cares for over 3500 hospitalized infants annually, with approximately 2400 of those children 6 months and younger of age. Notably, the AAP infant sleep policy does not address care for hospitalized infants; however, the hospital setting provides an opportunity to model safe sleep, provide education, and potentially improve safe sleep practices at home.^22–24^ Specifically, Krugman et al demonstrated a reduction in infant mortality through their efforts in a nursery setting.^25^ At baseline, our institution did not adhere to recommended safe sleep practices as ascribed by the AAP. However, the safe sleep task force (SSTF) noted the three particular drivers to change what we believed would improve our system, and we proceeded with interventions accordingly (Fig. 1).

### PROBLEM STATEMENT

Our institution showed poor adherence to safe sleep practices, with baseline data revealing a 75% weekly adherence to safe sleep recommendations indicating poor modeling of safe sleep behaviors. Through consistent education and modeling, inpatient adverse health events could be prevented, and implementation of safe sleep practices by caregivers could affect change on a larger community level.^14,25^

### SPECIFIC AIM STATEMENT

We aimed to increase the average weekly adherence to the AAP-recommended safe sleep practices for hospitalized infants at our institution to ≥95% over 12 months.

## METHODS

### CONTEXT

This institution is the only tertiary pediatric hospital in the state and serves as a regional referral center. It is a large pediatric enterprise with multiple organizational stakeholders, with the key stakeholder being the chief safety officer. The chief safety officer commissioned the SSTF to address the stated problem and comprises internal stakeholders, including nursing leadership, bedside nursing, nursing informatics, social work, occupational and physical therapists, and physicians. The Value Analysis committee of the hospital, which reviews requests for purchasing products, was also an internal stakeholder. We targeted interventions to three acute care medical-surgical floors responsible for caring for more than one-third of the approximately 3500 infants discharged annually.

### MEASUREMENT TOOL

**Fig. 2.**
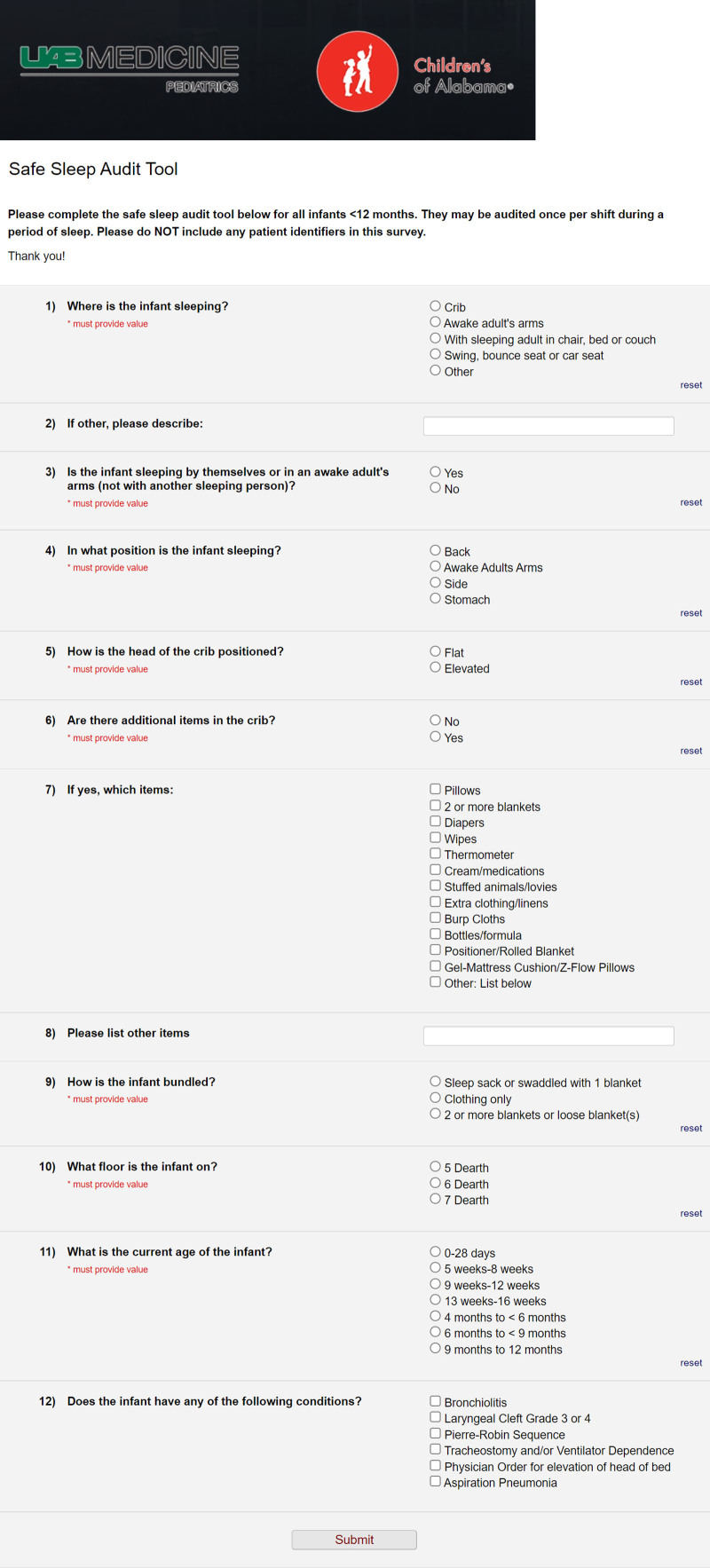
Safe Sleep Audit Tool. REDCap-based survey tool used by clinical assistant or nurse per hospitalized sleeping session to assess adherence to safe sleep guidelines. Red star denotes elements used for the calculation of adherence to guidelines.

Based on a review of the current literature, we created a crib audit to review adherence to the AAP’s recommendations. We adapted our tool from a previously published study because no nationally validated safe sleep audit tool exists for hospitalized infants.^13^ Six components of the recommendations that were applicable during hospitalization were the focus: location of sleep (crib, in arms, chair, couch), sleeping alone, the position of sleep (supine or prone), the elevation of the head of the bed, additional items in the crib, and bundling of the infant (Fig. 2). The correct components were: sleeping in a crib or an awake adult’s arms, sleeping alone, sleeping supine, flat head of the bed, no additional items in the crib, and bundled in only one blanket or sleep sack. Crib auditing began in October 2018 to obtain baseline adherence, and bedside nursing and clinical assistants (CAs) completed the audits. We included any observation of a sleep session for admitted infants on our three medical-surgical units without collecting protected health information. Individual patients were eligible for an audit once per nursing shift (8 hours) during a sleep session. There were no exclusion criteria for patients. Study data were collected and managed using Research Electronic Data Capture (REDCap). Institutional IRB deemed this project as a “Not Human Subjects Research.”

### INTERVENTIONS

#### Policy Change and Staff Education

We reviewed the current hospital policy on patient safety and compared it with the AAP recommendations. The existing policy lacked specificity and conviction. Therefore, the SSTF created a standalone Infant Safe Sleep Policy for all infants admitted to the hospital, excluding intensive care settings. Intensive care settings were excluded from some policy items, such as requiring supine positioning or flat head of the bed, due to the nature of critical care support provided, including mechanical ventilation. In addition, a nonadherence release form explicitly stating the risk of death was added to the policy for parents who repeatedly refused to adhere to the safe sleep policy.

We provided education to physicians through a Grand Rounds lecture and a didactic session to nursing staff, physical therapists, and occupational therapists outlining the importance of safe sleep recommendations and the efforts of the SSTF.

#### Parental Education

A parental education video was created and assigned as mandatory viewing for all parents of hospitalized infants. This video reviewed AAP recommendations for safe sleep and the expectation of inpatient sleep environments. The SSTF undertook initial efforts on education and policy change as we obtained baseline data.

#### Room Setup Change

**Fig. 3.**
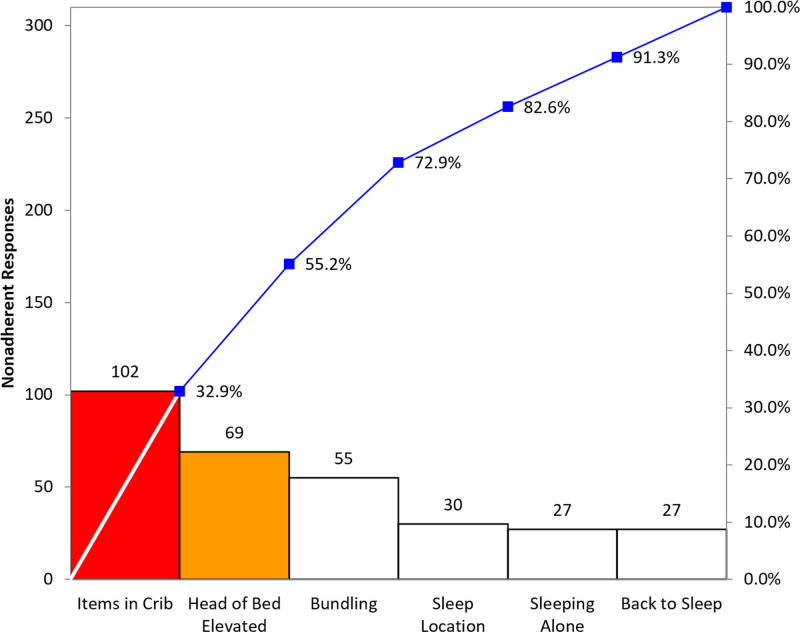
Pareto Chart of Baseline Categories with Greatest Nonadherence to Safe Sleep Guidelines. Review of baseline nonadherent responses revealed that greater than half of nonadherence to safe sleep guidelines was attributed to extra items in the crib and an elevated head of the bed for hospitalized infants. These data provided valuable insight into which categories to aim initial interventions.

A review of the data from the first 15 weeks revealed that extra items in the crib were the most significant contributor to nonadherence (Fig. 3).

After meeting with patient care staff, we utilized the Six Sigma 5S methodology to review the existing room set up for hospitalized infants.^26^ We created a new system to store extraneous items such as diapers, wipes, and creams commonly placed in the crib utilizing other surfaces and the available food cart, which was still easily accessible by staff.

#### Electronic Health Record Changes

The second highest nonadherence observation was the prevalence of the head of bed (HOB) elevation. Due to gastroesophageal reflux and respiratory disease concerns, many commonly used infant order sets had standing orders for HOB positioning. Literature review of North American Society for Pediatric Gastroenterology Hepatology, and Nutrition guidelines and discussions with institutional pediatric gastroenterologists were in line with AAP guidelines in stating that elevating the HOB is an ineffective treatment for infantile reflux and may result in respiratory compromise if the infant slides down the incline.^27^ Therefore, these standing orders were removed from the top 10 most commonly used infant order sets to deter nonadherence as part of initial buy-in to address the highest risk categories.^28^ Second, the order requiring the HOB to be elevated during enteral feeds was removed. These interventions were the first two steps leveraging the electronic health record (EHR) to address cultural beliefs and practice.

To provide additional clinical decision-making support in the EHR, we created a novel, safe sleep guideline and embedded it within infant admission order sets (**See figure, Supplemental Digital Content 1,** which shows the Electronic Health Record Safe Sleep Order Set. Detailed guidelines ordered upon admission for hospitalized infants were provided. Specific details were provided regarding the location and position of sleep as well as removal of extra items. Additionally, details regarding the use of newly implemented sleep sacks were provided, and education materials were reviewed. http://links.lww.com/PQ9/A344).

#### Sleep Sacks

**Fig. 4.**
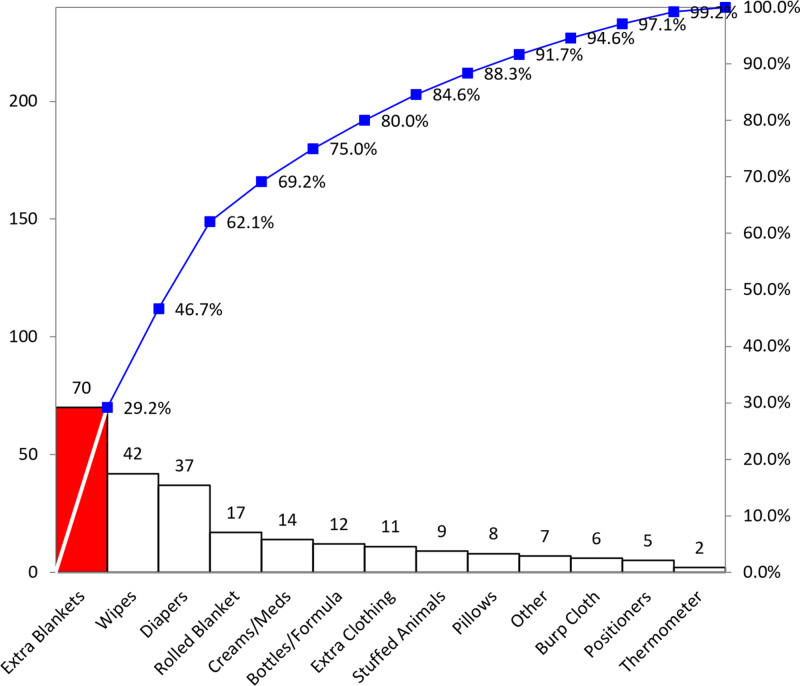
Pareto Chart of Extraneous Items in Crib. We analyzed specific extraneous items noted in the safe sleep survey audit to reveal that extra blankets, wipes, and diapers were the most commonly causing nonadherence to guidelines. This finding led to the adoption of diaper caddies to prevent these items from being placed in the crib.

After implementing policy and order set initiatives, PDSA cycles revealed that extraneous items in the crib continued to be an area of poor adherence. Upon reviewing items left in the crib, extra blankets contributed the most to nonadherence (Fig. 4). A safe sleep alternative in the form of sleep sacks, or wearable blankets, was identified. The hospital’s Value Analysis Committee approved the purchase of sleep sacks for hospitalized infants. With the assistance of an affiliated not-for-profit, the SSTF secured funding to give patients 6 months and younger a discharge gift of a sleep sack to reinforce the safe sleep education and behaviors beyond hospitalization.

#### Diaper Caddies

Wipes, diapers, and creams were the next most common nonadherent items from observations. To remove these items from the crib but still make them easily accessible, we purchased shower caddies to use as diaper caddies. We placed these safely outside the crib. The choice of diaper caddies (purchased from Dollar Tree, Chesapeake, Va.) to contain these items was based on success demonstrated *by* Geyer et al., and these were also gifted to families at discharge.

### MEASURES

We calculated two outcome measures tracking the six items queried on the crib audit (location of sleep, sleeping alone, sleep position, elevation of the head of the bed, additional items in the crib, and bundling of the infant). The outcomes measured were complete adherence to all six categories of safe sleep recommendations (yes = totally adherent and no = at least one nonadherent category) and percent adherence, giving 1-point credit for each adherent category with a denominator of 6 total points. Both complete adherence and percent adherence were recorded as a weekly average. Evaluation of each individual category using percent adherence was helpful. It allowed the SSTF to assess incremental improvements as we catered interventions to the most nonadherent categories (eg, inappropriate bundling and additional items in cribs). We also utilized complete adherence as it is the most common method used in the literature and creates a binary outcome of a safe (all six categories appropriate) or unsafe sleep environment.

### ANALYSIS

**Fig. 5.**
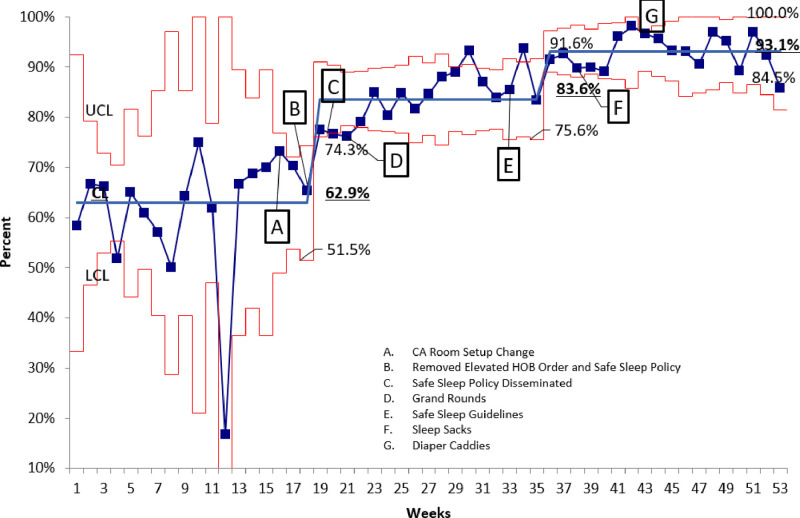
Annotated p-Chart of Average Weekly Adherence to Safe Sleep Guidelines. P-chart denoting the average percent adherence for each week for all children hospitalized on specific units. UCL = upper control limit set at 3 standard deviations above the mean and LCL = lower control limit set at 3 standard deviations below the mean. CL = center line, representing the average percent adherence per period. The average number is bold and underlined for the three different periods. Specific interventions annotated over time reveal a significant improvement in average weekly adherence from baseline weeks 1–18. We noted a trend shift greater than 8 points above the mean. Similarly, at week 35 when we implemented safe sleep guidelines in the electronic health records (Intervention E), we again noted an improvement in adherence to safe sleep guidelines, resulting in an overall 93.1% adherence to our safe sleep guidelines at the end of our project.

**Fig. 6.**
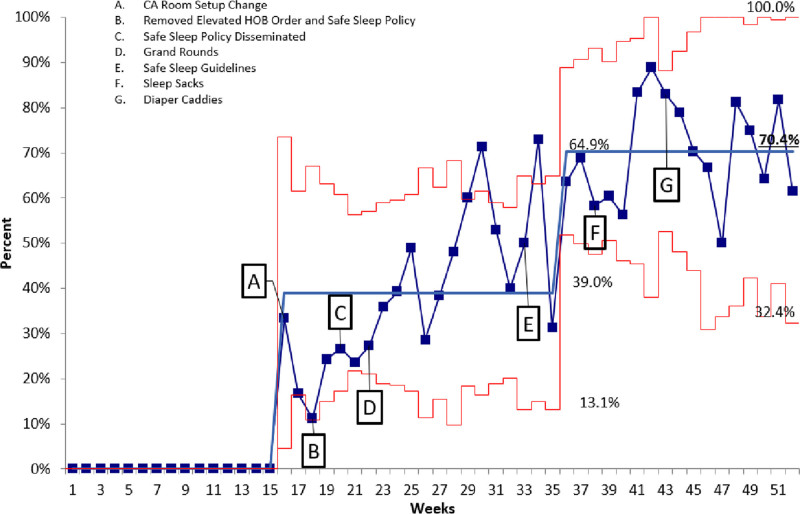
Annotated p-Chart of Average Weekly Completely Adherent Cribs. P-chart revealing the average weekly percent of cribs that were completely adherent to safe sleep guidelines. UCL= upper control limit set at 3 standard deviations above the mean and LCL = lower control limit set at 3 standard deviations below the mean. CL = center line, representing the average percent adherence per period. The average number is bold and underlined for the three different periods. Baseline data revealed that only <5% of cribs for hospitalized infants were entirely adherent to safe sleep guidelines. Over several weeks of interventions, we achieved a 70.4% complete adherence to safe sleep guidelines at the end of our efforts.

A proportions control chart utilizing the Institute for Healthcare Improvement (IHI) rules for assessing special cause variation and trend changes was utilized (Figs. 5, 6). Three standard deviations were used for control limits. Once the data fit the criteria for a significant trend, data were re-analyzed to create a new mean and control limits. Process control charts and Student’s t-testing were completed using QIMacros 2016 (KnowWare International, Inc., Denver, Colo.).

## RESULTS

There were 1560 observations made with an average of 130 completed monthly. There were two improvement trends noted on both measures of adherence (Figs. 5, 6). Adherence to recommendations improved from baseline (M = 70.8%, SD = 21.6) to the final 10 weeks postintervention completion (M = 94.7%, SD 10.0) [t(427) = −15.1, *P* ≤ 0.001]. Additionally, complete adherence was significantly increased from baseline (M = 0%, SD = 0) to the final 10 weeks (M = 70.4%, SD = 46) [t(381) = −21.4, *P* ≤ 0.001].

We noted significant improvements in adherence following the first phase of interventions (weeks 16–22). This improvement occurred before the initiation of high-reliability interventions (EHR changes and sleep sacks). It reflected a change in the behaviors at our institution that led to an overall improvement in safe sleep adherence to 84%.

The final PDSA cycles (EHR changes, sleep sacks, and diaper caddies) resulted in significant improvements. Specifically, we implemented the EHR safe sleep guidelines during week 35. As a result, the final two interventions, the use of sleep sacks and diaper caddies, were associated with a sustained improved adherence to 93%.

We have had 91.6% and 93.6% adherence by category on two follow-up annual crib audits and 62.5% and 70% for complete adherence.

## DISCUSSION

In summary, over the 52 weeks of this project, we demonstrated a significant improvement in adherence to safe sleep recommendations for hospitalized infants. We leveraged multiple tools and a vested multidisciplinary team to improve adherence to AAP safe sleep recommendations.

### INTERPRETATION

We demonstrated a significant improvement over time to change policy and provide education to staff and families.^29^ Based upon the longitudinal nature of this project with multiple steps taken in concert with engaged stakeholders both from an administrative and frontline perspective, the results reflect a larger cultural change in addressing safe sleep environments for hospitalized infants. The significant initial improvement after education was not anticipated, leading us to postulate on the immeasurable effect of culture change. Our theory on improved culture of safety around safe sleep is further strengthened with continued high adherence to safe sleep environments on follow-up audits, demonstrating sustainability.

The highest cost we incurred during this project was the purchase of sleep sacks. Through the HALO In-Hospital Safe Sleep Modeling Program, the hospital receives a free quarterly allotment to supplement the amount we purchase quarterly. Our current receiving blankets have significant laundering costs. We hope to make the costs of laundering infant sleep sacks cost-neutral by ultimately decreasing the use of receiving blankets.

The most important aspect of this project was the modeling of safe sleep behaviors. This modeling is significant because instruction from a healthcare provider is associated with increased adherence to safe sleep practices at home.^22^ Other studies have noted the detrimental effects of modeling unsafe behaviors and the positive effect of witnessing an infant placed supine during a hospitalization.^23,24^ Continued engagement in QI projects such as this one throughout the hospital may lead to overall improved quality and patient safety.^30^ This has been noted previously in educational efforts leading to improved patient outcomes.^11,16,19,25^

Of the interventions discussed, policy changes are typically not viewed as reliable QI interventions. However, the literature supports that a statewide policy as a singular intervention improved overall safe sleep practices, and Lighter states that “procedures emanate directly from policies and are the specific methods employed to actualize policies in day-to-day operations.”^16,26^ Therefore, the task force felt that the systematic change achievable by policy changes was essential. Based on feedback from bedside staff, the policy had a positive impact on staff adherence to guidelines. Similarly, educational efforts are an essential part of QI despite the decreased reliability of intervention.^31^ We also demonstrate the value of utilizing Lean and Six Sigma methodology whenever assessing an environment or process to reduce waste and variation. These initial interventions (didactics, interactive education, audits, and feedback) were the groundwork that culminated in multiple effective interventions.^32^

Electronic health record interventions are essential as they can establish protocols that standardize care. Our study overcame the dogma of elevating the HOB for respiratory conditions and continuous enteral feeds, also noted in another institution implementing safe sleep practices.^13^ In our pursuit of being a high-reliability organization, we deferred to our institutional experts and established national guidelines.^29^ Ultimately, this allowed us to garner acceptance from frontline staff. The safe sleep protocol was an essential part of creating a standard of care in our institution.

Other studies likewise found extraneous blankets in 33%–77% of cribs.^11,33^ Based upon efforts from other studies with the same barrier, we pursued the use of a wearable blanket/sleep sack.^10^ This intervention systematically changed the method in which we bundled hospitalized infants. As a result, loose blankets were less available, which in turn decreased excessive blankets in cribs.

Finally, we noted critical behavioral changes regarding safe sleep. While we did not objectively assess culture change, scheduled feedback sessions with frontline staff revealed increased “buy-in” and enthusiasm. These persistent improvements in adherence indicate a shift in the culture at our institution.

### LIMITATIONS

Although the study has strengths, some limitations exist. First, the possibility of a Hawthorne effect exists as the same provider tasked with providing safe sleep environments also completed the audit. It is worth noting that infants 4 months and older of age may roll over to sleep prone in a developmentally appropriate manner; however, staff would lose credit for adherence (not sleeping on the back) because our audit did not account for age leading to under-reporting safe sleep adherence. For these reasons, we set target adherence at ≥95%. Further, our safe sleep audit is not a validated measuring system, although we made efforts to parallel sleep audits used in other studies to provide external validity.^14^

As mentioned above, there is limited understanding of which particular interventions created the most impactful change and may overlap. Furthermore, given the unpredictable nature of SUID events on an annual basis, we cannot trend a clinical outcome by studying the impact on sleep-related deaths at the community or hospital level.

Third, we did not include parents as an essential stakeholder in our efforts and believe they would have provided a critical viewpoint.

Finally, this project aimed to address parts of the AAP safe sleep recommendations applicable to hospitalized infants. Notably, the AAP guidelines do not specifically address the inpatient setting and thus may not be valid for this patient population. In addition, this project did not address recommendations associated with other risk factors as they were outside this project’s scope, including avoidance of tobacco product exposure, pre- and postnatal drug use, breastfeeding, environmental risks such as cords. Thus, this project lacks generalizability to the outpatient setting.

### NEXT STEPS AND RECOMMENDATIONS

As part of the goal to improve outcomes for the local community, the institution has recently begun identifying patients during the admission process who do not have an appropriate sleep surface available at home. This intervention has initiated a standardized screening upon admission that triggers a social work consultation for patients without a crib, portable crib, or bassinet. Social workers are then able to provide a portable crib at the time of discharge. This effort highlights the importance of providing resources to underserved families and supporting postdischarge safe sleep adherence.

The SSTF will next implement these successful interventions across the entire hospital. As we spread our efforts, we plan to include parents as essential stakeholders in our task force. Furthermore, we hope to broaden our scope of education to include all hospital workers. Additionally, we hope to assess sustained safe sleep behaviors after discharge through the use of postdischarge questionnaires.

The financial impact of our endeavors was significant. Thus, a total return on investment analysis is planned to prove feasibility.

Finally, as part of this project, the task force applied for National Safe Sleep Hospital Certification through Cribs for Kids, which we obtained after completion. National recognition is an essential part of continued success and administrative support.

## CONCLUSION

The careful use of multiple QI modalities through a multidisciplinary team can have meaningful improvements in patient care and culture change to promote safe sleep behaviors at a large tertiary care center.

## DISCLOSURE

The authors have no financial interest to declare in relation to the content of this article.

## ACKNOWLEDGMENTS

We would like to acknowledge the whole of our Safe Sleep Task Force that did not directly assist with the study but were vital members of the team that led to sustained improvements in our project. Amber Sones, PT, DPT; Amelia Jones, RN; Andrew Payne, RN; Aneshia Williams, RPSGT, CCSE; April Jackson, MSN; M. Ashlie Cassidy, MSN, RN; Ashley Tate, RN; Crystal Calvert, RN; Erica Battle, RN; Jennifer Piziak, MSN, RN, CPN; Joseph Chewning, MD; Karisa Grizzle, MD; Kimberly Barnett, RN, CPN; Margaret Clark, LICSW; Megan McCroskey, LMSW; NaKeisha Portis, RN; Pallavi Ghosh, MD, MPH; Payton McBryde, MS, OTR/L; Russell Alan Thomas; Shaundra Blakemore, MD; Spandana Induru, MD; Vickie Hickman, RN.

Presented at Pediatric Quality and Safety Day, Birmingham, AL (Zoom Virtual), October 2020; Pediatric Academic Societies Annual Meeting, Philadelphia, PA, May 2020; National Symposium for Healthcare Executives, November 2019, Birmingham, AL.

## Supplementary Material


