# Supplementary material for: Improving Adherence to Safe Sleep Guidelines for Hospitalized Infants at a Children’s Hospital
Source: Pediatr Qual Saf. 2022 Jan 21;7(1):e508. doi: 10.1097/pq9.0000000000000508 (PMC8782121; doi:10.1097/pq9.0000000000000508)
Supplement: Supplementary file 1 [file pqs-7-e508-s001.pdf]

Supplemental Figure. Electronic Health Record Safe Sleep Order Set

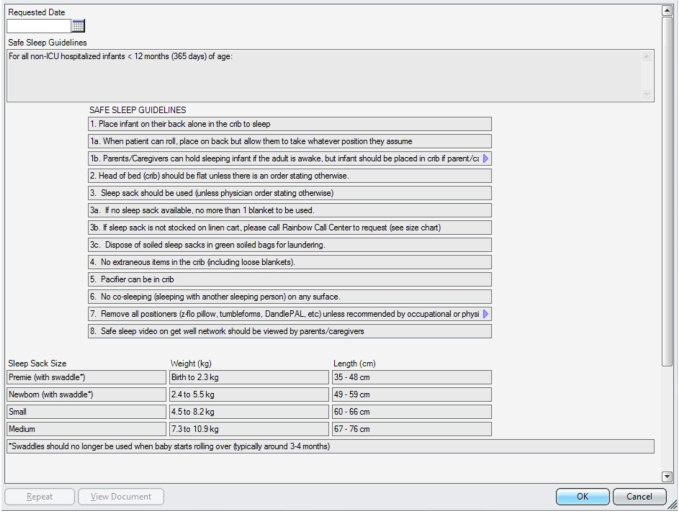

Requested Date

Safe Sleep Guidelines

For all non-ICU hospitalized infants < 12 months (365 days) of age:

SAFE SLEEP GUIDELINES

1. Place infant on their back alone in the crib to sleep
1a. When patient can roll, place on back but allow them to take whatever position they assume
1b. Parents/Caregivers can hold sleeping infant if the adult is awake, but infant should be placed in crib if parent/ca
2. Head of bed (crib) should be flat unless there is an order stating otherwise.
3. Sleep sack should be used (unless physician order stating otherwise)
3a. If no sleep sack available, no more than 1 blanket to be used.
3b. If sleep sack is not stocked on linen cart, please call Rainbow Call Center to request (see size chart)
3c. Dispose of soiled sleep sacks in green soiled bags for laundering.
4. No extraneous items in the crib (including loose blankets).
5. Pacifier can be in crib
6. No co-sleeping (sleeping with another sleeping person) on any surface.
7. Remove all positioners (z-flo pillow, tumbleforms, DandlePAL, etc) unless recommended by occupational or physi
8. Safe sleep video on get well network should be viewed by parents/caregivers

Sleep Sack Size	Weight (kg)	Length (cm)
Premie (with swaddle*)	Birth to 2.3 kg	35 - 48 cm
Newborn (with swaddle*)	2.4 to 5.5 kg	49 - 59 cm
Small	4.5 to 8.2 kg	60 - 66 cm
Medium	7.3 to 10.9 kg	67 - 76 cm

*Swaddles should no longer be used when baby starts rolling over (typically around 3-4 months)

Repeat | View Document | OK | Cancel
